# The Volatile Phytochemistry of *Monarda* Species Growing in South Alabama

**DOI:** 10.3390/plants10030482

**Published:** 2021-03-04

**Authors:** Sims K. Lawson, Prabodh Satyal, William N. Setzer

**Affiliations:** 1 Kirkland Gardens, P.O. Box 176, Newville, AL 36353, USA; skirkland.lawson@ufl.edu; 2 Aromatic Plant Research Center, 230 N 1200 E, Suite 100, Lehi, UT 84043, USA; psatyal@aromaticplant.org; 3 Department of Chemistry, University of Alabama in Huntsville, Huntsville, AL 35899, USA

**Keywords:** *Monarda citriodora*, *Monarda fistulosa*, *Monarda punctata*, essential oil, thymol, carvacrol, *p*-cymene

## Abstract

The genus *Monarda* (family Lamiaceae) contains 22 species of which three are native to southern Alabama, *M. citriodora*, *M. fistulosa*, and *M. punctata*. Several species of *Monarda* have been used in traditional medicines of Native Americans, and this present study is part of an ongoing project to add to our understanding of Native American pharmacopeia. Plant material from *M. citriodora, M. fistulosa*, and *M. punctata* was collected in south Alabama and the essential oils obtained by hydrodistillation. The essential oils were analyzed by gas chromatographic techniques to determine the chemical compositions as well as enantiomeric distributions. The compounds thymol, carvacrol, *p*-cymene, and their derivatives were the primary terpenoid components found in the essential oils. The known biological activities of these compounds are consistent with the traditional uses of *Monarda* species to treat wounds, skin infections, colds, and fevers.

## 1. Introduction

**Figure 1:**
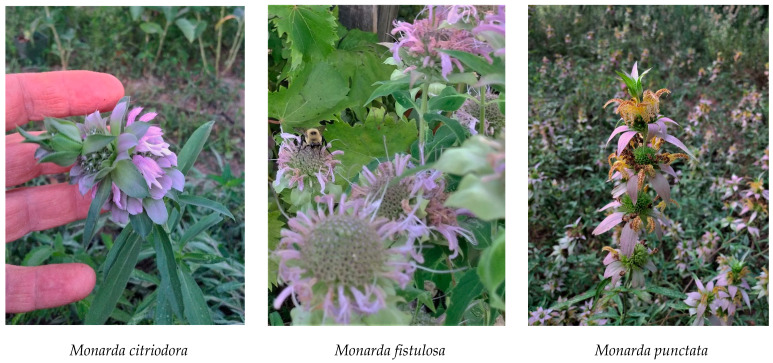
*Monarda* species discussed in this work (photographs by S. K. L).

The Plant List [1] shows 22 different *Monarda* L. (Lamiaceae) species, 18 of which occur in the United States [2]. There are three *Monarda* species native to south Alabama, namely *Monarda citriodora* Cerv. ex Lag., *Monarda fistulosa* L., and *Monarda punctata* L. (see Figure 1) [2].

Several *Monarda* species have been used by Native Americans as medicinal plants [3]. For example, *M. fistulosa* was used by the Blackfoot, Navajo, Lakota, and Winnebago people to treat boils, cuts and wounds; the Cherokee, Chippewa, Flathead, Ojibwa, and Tewa used the plant to treat colds, fever, and influenza; the Crow, Lakota, Menominee, and Ojibwa used the plant for coughs, catarrh, and other respiratory problems. *Monarda punctata* was used by the Delaware, Mohegan Nanticoke, and Navajo tribes to treat colds, fever coughs, and catarrh.

Both *M. citriodora* and *M. fistulosa* are popular ornamentals and have been introduced to temperate locations around the world [4,5,6]. Geographical location likely plays an important role in the phytochemistry of *Monarda* species. To our knowledge, however, there have been no previous examinations of *M. citriodora*, *M. fistulosa*, or *M. punctata* growing in their native range of south Alabama. In this work, we have examined the chemical compositions and enantiomeric distributions of essential oils of the three *Monarda* species from south Alabama.

## 2. Results

### 2.1. Monarda Citriodora

The *M. citriodora* essential oils were obtained as clear orange oils. The essential oil yields for *M. citriodora* aerial parts essential oil were 1.59% and 1.79% for samples #1 and #2, respectively, while the root essential oil was obtained in 0.879% yield. The chemical compositions of the essential oils from the aerial parts and the roots of *M. citriodora* cultivated in south Alabama are summarized in Table 1. The essential oils were dominated by the phenolic monoterpenoids thymol (RI_db_ = 1289) and carvacrol (RI_db_ = 1296). The other major components were *p*-cymene (RI_db_ = 1024) and thymol methyl ether (RI_db_ = 1239).

Chiral gas chromatography–mass spectrometry (GC-MS) analysis of the *M. citriodora* essential oils revealed the (+)-enantiomers to be the major stereoisomers for α-thujene, α-pinene, β-pinene, α-phellandrene, δ-3-carene, α-terpinene, *cis*-sabinene hydrate, *trans*-sabinene hydrate, α-terpineol, α-copaene, (*E*)-β-caryophyllene, and germacrene D. On the other hand, the (−)-enantiomer was dominant for β-phellandrene, borneol, carvone, and δ-cadinene. Limonene showed variation in the enantiomeric distributions with (+)-limonene in 26.7%, 63.3%, and 57.3% for aerial parts #1, #2, and roots essential oils, respectively. Likewise, linalool also showed variation with (+)-linalool of 71.9%, 49.7%, and 50.1%. (+)-Terpinen-4-ol was the predominant enantiomer in the aerial parts essential oils (60.2% and 58.5%), but (−)-terpinen-4-ol (79.1%) was dominant in the root essential oil.

### 2.2. Monarda Fistulosa

*Monarda fistulosa* essential oils were obtained in 2.66–4.83% yields as bright orange oils. The chemical compositions of the essential oils from the aerial parts of *M. fistulosa* are summarized in Table 2. In samples #1 and #2, thymol (RI_db_ = 1289) dominated the compositions (54.3% and 62.2%, respectively) with lesser quantities of *p*-cymene (RI_db_ = 1024, 12.1% and 10.2%), limonene (RI_db_ = 1030, 6.1% and 3.7%), carvacrol (RI_db_ = 1296, 5.9% and 6.6%), and thymoquinone (RI_db_ = 1252, 8.4% and 2.3%). Curiously, sample #3, although qualitatively similar, had a very different quantitative composition with thymoquinone as the most abundant constituent (41.3%) followed by *p*-cymene (21.9%), but with lower concentrations of thymol (8.9%) and carvacrol (1.6%).

As was observed in *M. citriodora* essential oils, in *M. fistulosa* essential oils, the (+)-enantiomer was the major for α-thujene, α-pinene, β-pinene, α-phellandrene, δ-3-carene, α-terpinene, *cis*-sabinene hydrate, *trans*-sabinene hydrate, α-terpineol, α-copaene, (*E*)-β-caryophyllene, and germacrene D, while the (−)-enantiomer was predominant for β-phellandrene and borneol. (−)-Limonene (97.4–99.5%) and (−)-linalool (62.1–62.5%) dominated in all three *M. fistulosa* samples. (+)-Camphene (100%), (+)-sabinene (58.4–59.0%), and (+)-terpinen-4-ol (63.2–63.3%) were also dominant.

### 2.3. Monarda Punctata

Hydrodistillation of two samples of wild-growing *M. punctata* aerial parts gave bright orange essential oils in 0.781% and 0.658% yield. The most abundant components in the essential oils were thymol (RI_db_ = 1289, 61.8% and 47.9%), *p*-cymene (RI_db_ = 1024, 15.3% and 19.8%), γ-terpinene (RI_db_ = 1057, 2.7% and 9.7%), and carvacrol (RI_db_ = 1296, 4.5% and 4.1%) (see Table 3).

The enantiomeric distributions of terpenoids in *M. punctata* essential oils were analogous to those observed for *M. citriodora* and *M. fistulosa* oils with the exception of limonene, which was virtually racemic in sample #1, but 100% (−)-limonene in sample #2.

## 3. Discussion

*Monarda citriodora* and *M. fistulosa* have been introduced throughout temperate regions of the world as popular herbal medicines as well as ornamentals [4,5,6]. The volatile phytochemistry has shown wide variation depending on geographical location (Table 4). The essential oils of *M. citriodora* in the present study were rich in both thymol and carvacrol, whereas essential oils from Europe and Asia were dominated by thymol with much lower concentrations of carvacrol. *Monarda fistulosa*, in particular, showed wide variation with at least three different chemotypes (carvacrol-rich, thymol-rich, and geraniol-rich, see Table 4). The essential oils of *M. fistulosa* (samples #1 and #2) in this study fit into the thymol-rich chemotype. Interestingly, there was a high concentration of thymoquinone in *M. fistulosa* sample #3, with concomitant lower concentrations of thymol and carvacrol. Thymol was reported as the major component of *M. punctata* in two old reports [11,12]. Consistent with these reports, a floral essential oil of *M. punctata* from China was rich in thymol (75.2%), which is in agreement with the aerial parts essential oils from Alabama.

The high concentrations of thymol, carvacrol, and *p*-cymene are consistent with the traditional uses of *Monarda* spp. to treat skin infections, wounds, fevers, and respiratory problems. Thymol [31], carvacrol [32], and *p*-cymene [33] have demonstrated antibacterial and antifungal activities [34,35], as well as wound-healing activity [36]. Thymol [37] and carvacrol [38], in addition to thymoquinone [39], have shown antitussive effects. Thymoquinone has also shown wound-healing properties [40]. Furthermore, both thymol [41] and carvacrol [32] have shown analgesic and anti-inflammatory activities [42].

As far as we are aware, this work presents the first chiral analysis of terpenoid constituents of *Monarda* species. Several investigations on the enantiomeric distributions in other members of the Lamiaceae have been reported in the literature, however. There seems to be much variation in the enantiomeric distribution of monoterpenoids across the family. Consistent with what was observed in *Monarda* essential oils, (+)-α-pinene was the major enantiomer found in *Coridothymus capitatus* [43], *Rosmarinus officinalis* [44], *Lepechinia heteromorpha* [45], *Ocimum canum*, and *Ocimum kilimandscharicum* [46]. Likewise, (+)-β-pinene predominates over (−)-β-pinene in *C. capitatus* [43] as well as the *Monarda* essential oils. On the other hand, (−)-β-pinene dominates in *R. officinalis* [44] and *Lepechinia mutica* [47]. The essential oils of peppermint (*Mentha* × *piperita*) and spearmint (*Mentha spicata*) have shown nearly racemic mixtures of α- and β-pinenes [48]. (+)-α-Phellandrene and (−)-β-phellandrene were the dominant enantiomers in the *Monarda* essential oils. In marked contrast, however, (−)-α-phellandrene and (+)-β-phellandrene predominated in *L. mutica* essential oil [47]. (−)-Limonene predominates in *M. fistulosa* essential oil, peppermint (*M. piperita*) and spearmint (*M. spicata*) essential oils [48] whereas (+)-limonene is the major enantiomer in *C. capitatus* [43], *O. canum*, and *O. kilimandscharicum* [46], and a nearly racemic mixture was found in rosemary (*R. officinalis*) essential oil [44]. (+)-Linalool was the predominant enantiomer in *C. capitatus* [43], *Salvia schimperi* [49], *Pycnanthemum incanum* [50], *O. canum*, and *O. kilimandscharicum* [46], whereas (−)-linalool was the major stereoisomer in *Lavandula angustifolia* [51] and *R. officinalis* [44].

## 4. Materials and Methods

### 4.1. Plant Material

*Monarda citriodora* was cultivated in Kirkland Gardens, Newville, AL, USA (31°26′27″ N, 85°21′31″ W) from seeds (Outsidepride Seed Source, Independence, OR, USA). The cultivated *Monarda* spp. were grown in loamy clayey-sand and fertilized with chicken manure, kelp meal, and bone meal at planting in full sun. The aerial parts of *M. citriodora* were collected from separate plants on separate occasions (plant #1, collected on 20 June 2020; plant #2 collected on 1 August 2020). The roots of *M. citriodora* were obtained from plant #2.

*Monarda fistulosa* was cultivated in Kirkland Gardens, Newville, AL, USA (31°26′27″ N, 85°21′31″ W) from seedlings (Home Depot, Dothan, AL, USA) as above. The aerial parts of three different plant samples were collected on 25 June 2020.

*Monarda punctata* was collected from wild-growing plants near Newville, AL, USA (31°27′23″ N, 85°22′17″ W); the edge of a planted pine forest, disturbed grassland, full/partial sun, sandy-clay soil that had been intentionally burned (prescribed burn) 1.5 years before collection. The aerial parts of two different plants were collected on 1 June 2020.

Plants were identified by S.K. Lawson and a voucher specimen of each plant was deposited in the University of Alabama in Huntsville Herbarium (HALA); voucher numbers for *M. citriodora* (SKL61820), *M. fistulosa* (SKL72020), and *M. punctata* (SKL9620). The *Monarda* plant materials were allowed to dry in the shade for several days, the air-dried plant materials were pulverized and subjected to hydrodistillation using a Likens-Nickerson apparatus with continuous extraction with dichloromethane (Table 5).

### 4.2. Gas Chromatographic Analysis

The essential oils were analyzed by gas chromatography–mass spectrometry (GC-MS), gas chromatography with flame ionization detection (GC-FID), and chiral GC-MS as previously reported [52].

#### 4.2.1. Gas Chromatography–Mass Spectrometry

Shimadzu GCMS-QP2010 Ultra, ZB-5ms GC column, GC oven temperature 50 °C–260 °C (2 °C/min), 1-μL injection of 5% solution of EO in dichloromethane (split mode, 30:1). Retention indices (RIs) were determined with reference to a homologous series of *n*-alkanes. Compounds identified by comparison of the MS fragmentation and retention indices with those in the databases [7,8,9,10].

#### 4.2.2. Gas Chromatography–Flame Ionization Detection

Shimadzu GC 2010, FID detector, ZB-5 GC column, GC oven temperature 50 °C–260 °C (2.0 °C/min). The percent compositions were determined from raw peak areas without standardization.

#### 4.2.3. Chiral Gas Chromatography–Mass Spectrometry

Shimadzu GCMS-QP2010S, Restek B-Dex 325 column, GC oven temperature 50 °C–120 °C (1.5 °C/min) then 120 °C–200 °C (2.0 °C/min), 0.1 μL injection of 5% solution of EO in dichloromethane (split mode, 45:1). The enantiomeric distributions were determined by comparison of retention times with authentic samples obtained from Sigma-Aldrich (Milwaukee, WI, USA). Relative enantiomer percentages were calculated from peak areas.

## 5. Conclusions

This study presents, for the first time, analyses of the essential oils of three species of *Monarda* growing in south Alabama. In addition, the enantiomeric distribution of terpenoids was also carried out. This work illustrates the wide variation in essential oil compositions based on geographical location as well as variations in enantiomeric distribution. It would be interesting to compare enantiomeric distributions for *Monarda* essential oils from other geographical locations and for other *Monarda* species. Nevertheless, the phenolic monoterpenoids thymol and/or carvacrol were found to dominate the compositions of *M. citriodora*, *M. fistulosa*, and *M. punctata* and support the traditional medicinal uses of these plants.

## Figures and Tables

**Table 1:** Essential oil compositions of *Monarda citriodora* cultivated in south Alabama.

			Aerial Parts Essential Oil				Root Essential Oil	
RI_calc_	RI_db_	Compound	#1, %	ED, (+):(−)	#2, %	ED, (+):(−)	#2, %	ED, (+):(−)
923	925	α-Thujene	1.0	69.0:31.0	0.8	66.6:33.4	0.5	64.2:35.8
930	932	α-Pinene	0.3	84.2:15.8	0.3	62.8:37.2	0.2	74.5:25.5
947	950	Camphene	tr		tr		tr	
970	971	Sabinene	tr		tr		---	
975	978	β-Pinene	0.1	64.5:35.5	0.1		0.1	
976	974	1-Octen-3-ol	0.7		0.7		0.5	
983	984	3-Octanone	0.1		0.2		0.3	
987	989	Myrcene	0.7		0.4		0.3	
995	996	3-Octanol	0.2		0.3		0.3	
1002	1004	Octanal	tr		tr		---	
1003	1004	*p*-Mentha-1(7),8-diene	tr		---		---	
1005	1006	α-Phellandrene	0.1	95.1:4.9	0.1	100:0	tr	100:0
1007	1008	δ-3-Carene	0.1	100:0	0.1	100:0	0.1	100:0
1015	1017	α-Terpinene	1.8	100:0	1.1	100:0	0.7	100:0
1016	1022	*m*-Cymene	tr		tr		---	
1023	1024	*p*-Cymene	7.8		6.4		7.2	
1027	1030	Limonene	0.5	26.7:73.3	0.4	63.3:36.7	0.3	57.3:42.7
1028	1029	β-Phellandrene	0.2	0:100	0.1	0:100	0.1	0:100
1030	1033	Benzyl alcohol	---		---		0.4	
1030	1030	1,8-Cineole	0.2		0.3		0.2	
1055	1057	γ-Terpinene	1.7		0.5		0.2	
1067	1069	*cis*-Sabinene hydrate	0.8	95.3:4.7	1.0	89.5:10.5	0.9	90.7:9.3
1083	1086	Terpinolene	tr		tr		tr	
1084	1086	*trans*-Linalool oxide (furanoid)	---		tr		0.1	
1087	1093	*p*-Cymenene	---		---		tr	
1098	1099	Linalool	0.1	71.9:28.1	0.1	49.7:50.3	0.3	50.1:49.9
1099	1099	*trans*-Sabinene hydrate	tr	76.5:23.5	0.3	71.1:28.9	0.3	66.8:33.2
1165	1167	*exo*-Acetoxycamphene	---		---		0.1	
1169	1170	Borneol	0.1	0:100	0.1	0:100	0.3	0:100
1178	1180	Terpinen-4-ol	0.4	60.2:39.8	0.4	58.5:41.5	1.9	20.9:79.1
1183	1186	*p*-Cymen-8-ol	---		---		0.1	
1187	1190	Methyl salicylate	tr		---		---	
1195	1195	α-Terpineol	0.1	100:0	0.1	100:0	0.2	100:0
1196	1197	Methyl chavicol (= Estragole)	---		1.5		---	
1236	1239	Thymol methyl ether	4.4		5.6		11.3	
1252	1252	Thymoquinone	0.2		0.7		1.3	
1253	1246	Carvone	tr	39.9:60.1	---		---	
1290	1289	Thymol	38.2		37.0		29.0	
1297	1296	Carvacrol	38.3		39.9		38.3	
1305	1309	4-Vinylguaiacol	---		---		0.1	
1306	1306	*iso*-Ascaridole	tr		tr		---	
1342	1345	Thymyl acetate	0.3		0.2		0.3	
1347	1356	Eugenol	tr		---		0.3	
1361	1365	Carvacryl acetate	0.8		0.5		1.0	
1372	1375	α-Copaene	tr	100:0	tr		0.1	100:0
1380	1382	β-Bourbonene	tr		tr		0.1	
1389	1392	(*Z*)-Jasmone	tr		tr		tr	
1398	1398	Cyperene	---		---		0.2	
1404	1408	Decyl acetate	tr		---		---	
1415	1417	(*E*)-β-Caryophyllene	0.3	100:0	0.4	100:0	0.5	100:0
1426	1430	β-Copaene	tr		tr		tr	
1451	1453	α-Humulene	tr		tr		tr	
1457	1457	Rotundene	---		---		0.1	
1471	1475	γ-Muurolene	tr		0.1		0.1	
1473	1481	(*E*)-β-Ionone	---		---		tr	
1477	1480	Germacrene D	0.1	100:0	0.1	100:0	0.1	
1481	1485	γ-Thujaplicin	tr		---		0.1	
1483	1489	β-Selinene	tr		---		---	
1487	1490	γ-Amorphene	tr		---		---	
1491	1497	α-Selinene	tr		tr		0.1	
1494	1497	α-Muurolene	tr		tr		tr	
1509	1512	γ-Cadinene	tr		tr		0.1	
1514	1518	δ-Cadinene	0.1		0.1		0.1	0:100
1548	1549	Thymohydroquinone	0.3		0.1		0.1	
1577	1577	Caryophyllene oxide	tr		0.1		0.1	
1649	1655	α-Cadinol	---		---		0.1	
1689	1691	Cyperotundone	---		---		0.2	
1835	1841	Phytone	---		---		0.1	
		Monoterpene hydrocarbons	14.3		10.2		9.6	
		Oxygenated monoterpenoids	84.0		86.3		86.0	
		Sesquiterpene hydrocarbons	0.5		0.7		1.4	
		Oxygenated sesquiterpenoids	tr		0.1		0.4	
		Benzenoid aromatics	tr		1.5		0.8	
		Others	1.0		1.2		1.2	
		Total identified	99.8		99.8		99.3	

RI_calc_ = Retention indices determined with respect to a homologous series of *n*-alkanes on a ZB-5ms column. RI_db_ = Retention indices from the databases [7,8,9,10]. #1 = Plant sample #1. #2 = Plant sample #2. --- = Not observed. ED = Enantiomeric distribution (dextrorotatory enantiomer: levorotatory enantiomer). tr = Trace (< 0.05%).

**Table 2:** Chemical composition of *Monarda fistulosa* essential oils cultivated in south Alabama.

			Aerial Parts Essential Oil					
RI_calc_	RI_db_	Compound	#1, %	ED, (+):(−)	#2, %	ED, (+):(−)	#3, %	ED, (+):(−)
923	925	α-Thujene	1.2	72.5:27.5	0.8	72.8:27.2	0.9	71.2:28.8
930	932	α-Pinene	0.5	59.2:40.8	0.3	63.8:36.2	0.5	61.0:39.0
947	950	Camphene	0.1	100:0	0.1	100:0	0.2	100:0
971	971	Sabinene	0.2	58.4:41.6	tr		0.2	59.0:41.0
973	973	1-Octen-3-one	---		---		0.1	
975	978	β-Pinene	0.2	57.3:42.7	---		0.2	57.9:42.1
978	978	1-Octen-3-ol	3.0		3.3		3.3	
982	984	3-Octanone	tr		0.1		0.1	
987	989	Myrcene	tr		0.3		0.1	
995	996	3-Octanol	tr		0.1		0.1	
1004	1004	*p*-Mentha-1(7),8-diene	tr		tr		tr	
1006	1006	α-Phellandrene	0.1	95.5:4.5	0.2	95.4:4.6	0.1	93.4:6.6
1008	1008	δ-3-Carene	0.1	100:0	0.1	100:0	0.1	100:0
1016	1017	α-Terpinene	2.1	100:0	2.3	100:0	0.8	100:0
1019	1022	*m*-Cymene	tr		tr		0.1	
1024	1024	*p*-Cymene	12.1		10.2		21.9	
1025	1026	2-Acetyl-3-methylfuran	---		---		0.5	
1029	1030	Limonene	6.1	0.5:99.5	3.7	2.6:97.4	6.3	1.2:98.8
1030	1031	β-Phellandrene	0.2	0:100	0.2	0:100	0.2	0:100
1031	1030	1,8-Cineole	0.1		0.1		0.1	
1056	1057	γ-Terpinene	tr		0.1		tr	
1069	1069	*cis*-Sabinene hydrate	1.2	95.8:4.2	1.3	96.3:3.7	2.4	96.5:3.5
1078	1079	1-Nonen-3-ol	0.1		0.1		0.1	
1084	1086	Terpinolene	tr		0.1		tr	
1089	1091	*p*-Cymenene	tr		tr		0.1	
1098	1099	Linalool	tr	37.8:62.2	tr	37.5:62.5	tr	37.9:62.1
1099	1099	*trans*-Sabinene hydrate	0.2	75.9:24.1	0.3	75.0:25.0	0.5	75.3:24.7
1103	1107	Nonanal	tr		---		tr	
1115	1112	(*E*)-2,4-Dimethylhepta-2,4-dienal	---		---		0.2	
1121	1121	*trans-p*-Mentha-2,8-dien-1-ol	tr		tr		0.3	
1123	1124	*cis-p*-Menth-2-en-1-ol	tr		tr		tr	
1130	1132	*cis*-Limonene oxide	tr		---		0.1	
1133	1135	2-Vinylanisole	0.1		tr		tr	
1134	1137	*cis-p*-Mentha-2,8-dien-1-ol	---		---		0.3	
1135	1138	*trans*-Limonene oxide	tr		---		---	
1137	1138	*trans*-Sabinol	---		---		tr	
1138	1140	*trans*-Pinocarveol	---		---		0.1	
1139	1141	*cis*-Verbenol	---		---		tr	
1143	1145	*trans*-Verbenol	---		---		0.3	
1144	1145	Camphor	---		---		tr	
1160	1164	Pinocarvone	---		---		tr	
1161	1162	(*Z*)-*iso*-Citral	---		---		tr	
1167	1168	*trans*-Phellandrene epoxide	---		---		0.1	
1170	1170	Borneol	0.5	0:100	0.2	0:100	0.7	0:100
1179	1180	Terpinen-4-ol	0.4	63.3:36.7	0.5	63.2:36.8	0.5	
1186	1186	*p*-Cymen-8-ol	0.1		tr		0.6	
1195	1195	α-Terpineol	0.3	100:0	0.2	100:0	0.3	
1197	1198	Methylchavicol (= Estragole)	---		0.1		0.1	
1197	1198	*cis*-Piperitol	---		---		0.2	
1217	1218	*trans*-Carveol	---		---		0.2	
1231	1232	*cis*-Carveol	---		---		0.1	
1240	1242	Cuminaldehyde	---		---		0.1	
1241	1242	Carvone	---		---		0.3	
1250	1241	Pulegone	---		0.2		---	
1252	1252	Thymoquinone	8.4		2.3		41.3	
1281	1282	Bornyl acetate	---		---		0.1	
1284	1286	Cogeijerene	0.1		---		---	
1291	1291	*p*-Cymen-7-ol	tr		---		0.2	
1295	1293	Thymol	54.3		62.2		8.9	
1300	1300	Carvacrol	5.9		6.6		1.6	
1307	1306	*iso*-Ascaridole	tr		tr		0.1	
1345	1346	α-Cubebene	tr		tr		tr	
1351	1356	Eugenol	tr		tr		---	
1373	1375	α-Copaene	0.1	100:0	0.1	100:0	0.1	100:0
1382	1382	β-Bourbonene	0.1		0.1		0.1	
1387	1387	*trans*-β-Elemene	tr		tr		0.1	
1418	1419	β-Ylangene			tr		0.1	
1419	1417	(*E*)-β-Caryophyllene	0.3	100:0	0.3	100:0	0.2	100:0
1427	1430	β-Copaene	0.1		0.1		0.1	
1452	1453	α-Humulene	tr		tr		tr	
1473	1475	γ-Muurolene	0.1		0.2		0.1	
1479	1479	α-Amorphene	---		tr		---	
1480	1483	*trans*-β-Bergamotene	0.1		---		0.1	
1481	1480	Germacrene D	0.7	100:0	0.6	100:0	0.6	100:0
1484	1485	γ-Thujaplicin	0.4		0.2		1.3	
1488	1490	γ-Amorphene	tr		---		---	
1491	1492	β-Selinene	0.1		0.1		0.1	
1493	1492	*trans*-Muurola-4(14),5-diene	---		0.1		---	
1496	1497	*epi*-Cubebol	---		tr		---	
1497	1497	α-Selinene	---		0.1		---	
1498	1497	α-Muurolene	tr		0.1		tr	
1510	1512	γ-Cadinene	0.1		0.2		0.1	
1511	1515	Cubebol	tr		---		---	
1517	1518	δ-Cadinene	0.1		0.3		0.1	
1518	1519	*trans*-Calamenene	tr		tr		---	
1520	1523	β-Sesquiphellandrene	tr		---		---	
1537	1538	α-Cadinene	tr		tr		---	
1542	1541	α-Calacorene	tr		tr		---	
1543	1546	α-Elemol	tr		---		---	
1548	1554	Thymohydroquinone	0.6		1.6		0.6	
1558	1565	Eugenyl acetate	tr		tr		tr	
1559	1557	Germacrene B	tr		---		---	
1580	1577	Caryophyllene oxide	tr		tr		0.1	
1638	1639	*cis*-Guaia-3,9-dien-11-ol	0.1		0.1		---	
1651	1655	α-Cadinol	tr		0.1		tr	
		Monoterpene hydrocarbons	22.8		18.3		31.4	
		Oxygenated monoterpenoids	72.2		75.7		61.2	
		Sesquiterpene hydrocarbons	1.7		2.2		1.7	
		Oxygenated sesquiterpenoids	0.1		0.1		0.1	
		Benzenoid aromatics	0.1		0.1		0.1	
		Others	3.1		3.6		4.3	
		Total identified	100.0		100.0		98.7	

RI_calc_ = Retention indices determined with respect to a homologous series of *n*-alkanes on a ZB-5ms column. RI_db_ = Retention indices from the databases [7,8,9,10]. #1 = Plant sample #1. #2 = Plant sample #2. #3 = Plant sample #3. --- = Not observed. ED = Enantiomeric distribution (dextrorotatory enantiomer: levorotatory enantiomer). tr = Trace (<0.05%).

**Table 3:** Chemical composition of *Monarda punctata* essential oils growing wild in south Alabama.

			Aerial Parts Essential Oil			
RI_calc_	RI_db_	Compound	#1, %	ED, (+):(−)	#2, %	ED, (+):(−)
923	925	α-Thujene	0.1	100:0	0.7	68.5:31.5
930	932	α-Pinene	tr		0.2	83.8:16.2
945	950	Camphene	---		0.1	100:0
957	959	Benzaldehyde	---		tr	
970	971	Sabinene	tr		tr	
974	978	β-Pinene	tr		0.1	62.7:37.3
975	974	1-Octen-3-ol	1.8		1.8	
980	983	3-Octanone	---		tr	
986	989	Myrcene	0.3		1.1	
995	996	3-Octanol	0.1		0.1	
1001	1004	*p*-Mentha-1(7),8-diene	---		tr	
1004	1006	α-Phellandrene	0.1	100:0	0.2	94.6:5.4
1006	1008	δ-3-Carene	tr		0.1	100:0
1014	1017	α-Terpinene	1.3	100:0	3.0	100:0
1016	1022	*m*-Cymene	---		tr	
1024	1024	*p*-Cymene	15.3		19.8	
1025	1026	2-Acetyl-3-methylfuran	---		tr	
1026	1030	Limonene	0.4	50.2:49.8	0.5	0:100
1027	1029	β-Phellandrene	0.2		0.2	0:100
1028	1030	1,8-Cineole	0.4		0.1	
1039	1043	Phenylacetaldehyde	---		tr	
1055	1057	γ-Terpinene	2.0		9.7	
1066	1069	*cis*-Sabinene hydrate	0.6	100:0	0.7	97.9:2.1
1076	1079	1-Nonen-3-ol	---		tr	
1082	1086	Terpinolene	0.1		0.1	
1087	1091	*p*-Cymenene	0.1		0.1	
1095	1099	Linalool	---		tr	70.1:29.9
1098	1101	*trans*-Sabinene hydrate	0.2	100:0	0.1	83.2:16.8
1100	1104	Nonanal	---		0.1	
1104	1107	1-Octen-3-yl acetate	0.2		0.4	
1145	1145	*trans*-Verbenol	---		tr	
1161	1158	Menthone	0.3		---	
1168	1170	Borneol	0.1	0:100	tr	0:100
1177	1180	Terpinen-4-ol	0.8	58.7:41.3	0.6	66.1:33.9
1182	1183	*m*-Cymen-8-ol	---		0.1	
1184	1186	*p*-Cymen-8-ol	0.5		0.5	
1191	1197	Methyl chavicol (= Estragole)	0.8		---	
1193	1195	α-Terpineol	---		0.1	100:0
1202	1206	Decanal	---		0.1	
1224	1224	Thymol methyl ether	---		tr	
1235	1238	Carvacrol methyl ether	1.1		1.0	
1239	1242	Cumin aldehyde	---		0.1	
1247	1250	Thymoquinone	2.0		0.2	
1289	1289	Thymol	61.8		47.9	
1293	1291	*p*-Cymen-7-ol	---		0.2	
1296	1296	Carvacrol	4.5		4.1	
1306	1309	4-Vinylguaicol	---		tr	
1347	1356	Eugenol	0.7		0.3	
1370	1375	α-Copaene	---		tr	
1378	1382	β-Bourbonene	---		tr	
1384	1390	*trans*-β-Elemene	---		tr	
1415	1417	(*E*)-β-Caryophyllene	1.6	100:0	1.2	100:0
1424	1430	β-Copaene	---		tr	
1427	1430	*trans*-α-Bergamotene	1.2		0.7	
1449	1453	α-Humulene	---		tr	
1469	1475	γ-Muurolene	---		0.1	
1476	1480	Germacrene D	0.7	100:0	0.4	100:0
1479	1483	*trans*-β-Bergamotene	0.2		0.1	
1480	1485	γ-Thujaplicin	---		0.1	
1482	1489	β-Selinene	---		tr	
1489	1492	α-Selinene	---		tr	
1492	1497	α-Muurolene	---		tr	
1506	1512	γ-Cadinene	---		tr	
1512	1518	δ-Cadinene	---		0.1	
1517	1523	β-Sesquiphellandrene	---		0.1	
1546	1549	Thymohydroquinone	0.3		2.5	
1576	1577	Caryophyllene oxide	0.2		0.2	
1633	1639	*cis*-Guaia-3,9-dien-11-ol	0.2		0.1	
1648	1655	α-Cadinol	---		tr	
1834	1841	Phytone	---		tr	
		Monoterpene hydrocarbons	19.9		35.7	
		Oxygenated monoterpenoids	72.5		58.3	
		Sesquiterpene hydrocarbons	3.7		2.7	
		Oxygenated sesquiterpenoids	0.4		0.4	
		Benzenoid aromatics	1.5		0.3	
		Others	2.1		2.4	
		Total identified	100.0		99.8	

RI_calc_ = Retention indices determined with respect to a homologous series of *n*-alkanes on a ZB-5ms column. RI_db_ = Retention indices from the databases [7,8,9,10]. #1 = Plant sample #1. #2 = Plant sample #2. --- = Not observed. ED = Enantiomeric distribution (dextrorotatory enantiomer: levorotatory enantiomer). tr = Trace (<0.05%).

**Table 4:** Major essential oil components of *Monarda* species from geographical locations around the world.

*Monarda* spp.	Plant Tissue	Collection Site	Composition (Major Components)	Ref.
*M. citriodora*	Aerial parts	Jammu, India (cultivated)	Thymol (82.3%), carvacrol (4.8%)	[13]
*M. citriodora*	Aerial parts	Imola (BO) Italy (cultivated)	Thymol (19.6%), *p*-cymene (15.6%), γ-terpinene (13.5%), carvacrol (9.3%), α-terpinene (9.2%), myrcene (5.7%)	[14]
*M. citriodora*	Not reported	Commercial (India)	(*E*)-β-Caryophyllene (19.2%), citral ^a^ (13.3%), limonene (11.8%), *cis*-verbenol (11.4%), geraniol (7.6%), citronellal (5.6%)	[15]
*M. citriodora* var. *citriodora*	Leaves	Liverpool, UK (cultivated)	Thymol (50.7%), *p*-cymene (22.8%), carvacrol (3.6%)	[16]
*M. citriodora* var. *citriodora*	Flowers	Liverpool, UK (cultivated)	Thymol (61.8%), γ-terpinene (13.3%), *p*-cymene (4.2%), carvacrol (3.8%)	[16]
*M. citriodora* var. *citriodora*	Aerial parts	Liverpool, UK (cultivated)	Thymol (56.9%), *p*-cymene (13.0%), α-terpinene (10.0%), carvacrol (4.3%)	[17]
*M. citriodora* var. *citriodora*	Aerial parts	Commercial (unknown)	Thymol (70.6%), *p*-cymene (10.6%), carvacrol (6.1%)	[18]
*M. fistulosa*	Aerial parts	Krasnodarsk Krai, Russia (introduced, wild)	p-Cymene (32.5%), carvacrol (23.9%), thymol (12.6%), carvacrol methyl ether (5.5%), unidentified aliphatic aldehyde (6.3%)	[19]
*M. fistulosa*	Aerial parts	Casola Valsenio, Italy (cultivated)	Thymol (26.5%), β-phellandrene (17.0%), α-phellandrene (13.7%), *p*-cymene (13.5%), myrcene (8.1%)	[20]
*M. fistulosa*	Aerial parts	Saint-Jean-sur-Richelieu, QC, Canada (cultivated)	Geraniol (61.8%), geranyl formate (16.6%), geranial (10.6%), neral (6.6%)	[21]
*M. fistulosa*	Aerial parts	Poplarville, MS, USA (cultivated)	Carvacrol (39.1%), *p*-cymene (35.4%), (−)-1-octen-3-ol	[22]
*M. fistulosa*	Aerial parts	Imola (BO) Italy (cultivated)	Thymol (31.6%), β-phellandrene (18.1%), α-phellandrene (14.2%), *p*-cymene (13.1%), myrcene (8.8%)	[23]
*M. fistulosa*	Aerial parts	Imola (BO) Italy (cultivated)	Thymol (28.4%), β-phellandrene (16.9%), α-phellandrene (13.7%), *p*-cymene (13.3%), myrcene (8.7%)	[24]
*M. fistulosa*	Aerial parts	Imola (BO) Italy (cultivated)	Thymol (33.4%), β-phellandrene (18.0%), α-phellandrene (14.0%), *p*-cymene (13.2%), myrcene (8.6%)	[24]
*M. fistulosa*	Aerial parts	Ravenna, Italy (cultivated)	γ-Terpinene (25.2%), carvacrol (24.3%), *p*-cymene (11.0%; reported as *o*-cymene), thymol (8.4%), α-terpinene (5.0%), thymol methyl ether (4.7%)	[25]
*M. fistulosa*	Aerial parts	Chişinău, Republic of Moldova (cultivated)	Carvacrol (54.8%), *p*-cymene (23.2%), carvacrol methyl ether (5.9%)	[26]
*M. fistulosa*	Flowers	Gallatin Valley, MT, USA (wild)	Carvacrol (45.7%), *p*-cymene (25.6%), γ-terpinen (6.8%), thymol (3.1%)	[27]
*M. fistulosa*	Leaves	Gallatin Valley, MT, USA (wild)	Carvacrol (71.5%), *p*-cymene (13.1%), γ-terpinen (2.5%), thymol (3.3%)	[27]
*M. fistulosa*	Aerial parts	Moscow, Russia (cultivated)	α-Terpineol (37.7%), 1-octen-3-ol (10.5%), geraniol (10.4%), thymol (9.3%), *p*-cymene (4.9%)	[28]
*M. fistulosa* cv. Fortuna	Aerial parts	Kherson, Ukraine (cultivated)	Thymol (77.3%), carvacrol methyl ether (4.9%), carvacrol (3.8%)	[6]
*M. fistulosa* cv. Premiera	Aerial parts	Kherson, Ukraine (cultivated)	Thymol (78.3%), carvacrol methyl ether (4.8%), carvacrol (3.6%)	[6]
*M. fistulosa* var. *menthifolia*	Aerial parts	Morden, Manitoba, Canada (cultivated)	Geraniol (86.8%)	[29]
*M. punctata*	Flowers	Xi’an, China (cultivated?)	Thymol (75.2%), *p*-cymene (6.7%), limonene (5.4%), carvacrol (3.5%)	[30]

^a^ Isomer not indicated.

**Table 5:** Hydrodistillation details of *Monarda* species collected or cultivated in south Alabama.

*Monarda* spp.	Mass Plant Material	Yield Essential Oil (EO)
*Monarda citriodora* #1	25.57 g dried aerial parts	406.2 mg orange EO
*Monarda citriodora* #2	37.81 g dried aerial parts	675.6 mg orange EO
*Monarda citriodora* #2	17.47 g dried roots	153.6 mg yellow EO
*Monarda fistulosa* #1	9.60 g dried aerial parts	364.0 mg bright orange EO
*Monarda fistulosa* #2	7.58 g dried aerial parts	366.2 mg bright orange EO
*Monarda fistulosa* #3	8.98 g dried aerial parts	238.9 mg bright orange EO
*Monarda punctata* #1	39.09 g dried aerial parts	305.6 mg bright orange EO
*Monarda punctata* #2	62.62 g dried aerial parts	411.9 mg bright orange EO

## Data Availability

All data are contained within the article.
